# Effect of the use of probiotic *Bacillus subtilis* (QST 713) as a growth promoter in broilers: an alternative to bacitracin methylene disalicylate

**DOI:** 10.1016/j.psj.2021.101372

**Published:** 2021-07-02

**Authors:** Walter Rivera-Pérez, Elías Barquero-Calvo, Aida J. Chaves

**Affiliations:** ⁎ Tropical Diseases Research Program, School of Veterinary Medicine, Universidad Nacional, Heredia 40104, Costa Rica; † Avian Pathology Laboratory, School of Veterinary Medicine, Universidad Nacional, Heredia 40104, Costa Rica

**Keywords:** probiotic, *Bacillus subtilis*, gut health, microbiota, broiler

## Abstract

The global poultry trend toward the more responsible use of antibiotics is becoming recurrent and has demanded the need to generate new natural alternatives. Probiotics have gained importance as an option to use as growth promoters. This study aimed to evaluate *Bacillus subtillis* QST713 as a substitute for an antibiotic growth promoter (**BMD**). A total of 150 male broilers were assigned to three dietary treatments: 1) control diet (**CO**), 2) control diet + 500 g/t of BMD (**AGP**), and 3) control diet + 100 g/t of *B. subtilis* QST713 (**PB**), respectively. Each treatment was monitored for 5 wk for the productive variables: body weight, accumulated feed consumption, food conversion, and European efficiency factor. At the end of each week, fresh fecal samples were cultured and quantified for *E. coli, Enterococcus* spp., and *Lactobacillus* spp. At the end of the trial, blood samples were analyzed for hemogram and intestinal samples (anterior portion) for histomorphometry. The data were statistically analyzed with an analysis of variance and subjected to a least significant difference test (Tukey). The zootechnical yields were similar in the AGP and PB groups (*P* ˃ 0.05); both superior to the control group. In the hematological profiles, no difference was observed between the experimental groups. *E. coli* and *Enterococcus* counts were significantly lower (*P* ˂ 0.05), and *Lactobacillus* counts were significantly (*P* ˂ 0.05) higher in the PB group, relative to CO and AGP groups. No differences (*P* ˃ 0.05) were found in bacterial counts between the CO and AGP groups. The intestinal mucosa and villi in the PB group were significantly (*P* ˂ 0.05) longer and with less deeper crypts than CO and AGP groups. We conclude that *B. subtillis* QST713, used at the suggested commercial dose (100 g/ton), is an effective growth-promoting alternative to BMD that modulates the microbiota and intestinal architecture, thus producing zootechnical yields consistent with BMD.

## INTRODUCTION

The world's human population continues to grow rapidly, and with it, the demand for animal protein increases, so the livestock industry must improve the productive performance of the animals (Bilal et al., 2021). Additionally, new demands also emerge from the consumer, for example, partial or total restrictions to the use of antibiotic growth promoters (**AGP**) (Bai et al., 2017). The current population shows great interest in food safety and security. In addition, many governments create laws and regulations that regulate the use of antibiotics (Zhang et al., 2021), so the poultry industry is focused on looking for alternatives to antibiotics that maintain the performance and health of animals in productive conditions (Ciurescu et al., 2020).

The AGP has been used in poultry feeds throughout the world in subtherapeutic concentrations and has been used for almost 8 decades (Oladokun et al., 2020). Bacitracin Methylene Disalicylate (**BMD**) is one of the most widely used, the benefits of its use date from the 50s (Branion et al., 1952) and apparently maintains its effectiveness. BMD inhibits certain intestinal bacteria and modulates the intestinal microflora (Engberg et al., 2000). BMD acts mainly on Gram-positive bacteria, interferes with their cell membrane's function, suppresses the formation of the cell wall, and inhibits protein synthesis (Sims et al., 2004).

Probiotics (**PB**) have been increasingly adopted as an alternative to AGP in poultry diets (Ciurescu et al., 2020). PB are a nutritional tool that has been shown to improve production rates and health in broilers, help prevent diseases, and improves infection recovery (Bilal et al., 2021). *B. subtilis*, a nonpathogenic spore-forming bacterium, has been considered one of the most successful probiotic bacteria in poultry nutrition due to its resistance to temperature change during the feed manufacturing process and long storage term, also supports extreme gastrointestinal environments with low pH (Manafi et al., 2017; Ciurescu et al., 2020).

Dietary supplementation with *B. subtilis* has positive effects that improve the performance of the birds by creating a favorable gut environment for an adequate intestinal microflora in the host, which translates into better feed conversion and digestive efficiency (Bai et al., 2017; Oladokun et al., 2020). The primary mode of action of *B. subtilis* spores is related to their ability to create an anaerobic environment in the intestine after germination. *B. subtilis* stimulates the growth and proliferation of native lactobacilli, which leads to competitive colonization and production of lactic acid. Ultimately, this results in restrictions for developing pathogenic bacteria in the host's intestines (Jeong and Kim 2014).

As described above, there is a growing concern about the use of AGPs due to their potential ability to induce cross-resistance for pathogenic bacteria for humans, which is why it is necessary to offer alternatives to production systems. To evaluate a nonantibiotic growth-promoting option, here we measure the supplementary efficacy of *B. subtilis* (QST713) as a natural growth promoter and their effect on 1) zootechnical parameters; 2) intestinal architecture; 3) hematological characteristics, and 4) modulation of the intestinal microflora. These effects were continuously monitored over time (5 wk) in each study group.

## MATERIALS AND METHODS

### Birds, Diet, and Experimental Design

The trial was conducted in the experimental farm of the Laboratory of Avian Pathology of the School of Veterinary Medicine of the National University of Costa Rica, Heredia, Costa Rica.

One hundred and fifty commercial 1-day-old male broilers (Cobb × Cobb) were purchased from a local commercial broiler hatchery, Rio Segundo, Alajuela, Costa Rica. Animals were randomly assigned into 3 treatments, composed of 5 replicates each. Each replica contained 10 birds for a total of 50 birds per treatment. A randomized complete blocks design with 5 blocks and 3 treatments per block was used.

The trial had 3 treatments: 1) control group (**CO**) fed commercial diets (2 stages); 2) antibiotic group (**AGP**) fed the diets mixed with BMD (Zoetis) at 500 g/ton; and 3) probiotic group (**PB**) fed with the diets mixed with a probiotic (*B. subtilis* QST 713, Grobig Bayer) at 100 g/ton. Both experimental treatments (AGP and PB) were used at the single doses recommended in their respective leaflets as growth promoters.

The study started when the birds were housed (d 1) in their respective treatment and replicated and fed ad libitum. The trial lasted 35 d, and the birds were housed in a cage system at a density of 10 birds/1.2 m^2^. The light was provided using the lighting programs suggested for the genetic line (Cobb Vantress, 2018).

The use, composition, and nutrients of the feed offered during the trial are shown in Table 1. The feed was formulated according to the nutritional requirements of the Cobb 500 broilers and prepared in a commercial feed manufacturing factory, as established in the Central American Technical Regulation RTCA 65.05.63: 13 Products used in animal nutrition.

**Table 1:** Use, composition, and nutrient content of the base feeding plan (two stages) offered to broilers.

Parameter	Starter	Grower
Days in use	1 a 21	22 a 35
Ingredients (%)		

Corn	57,77	59,15
Soya	33,00	31,00
Vegetal oil	3,60	4,50
DDG´S	2,90	2,80
Calcium carbonate	1,20	1,10
Monocalcium phosphate	1,08	1,00
Salt	0,30	0,30
Premix (vitamis+minerals)	0,15	0,15

Total	100,00	100,00

Nutrients		
Crude protein (%)	21,00	19,50
Lysine (%)	1,30	1,20
Methionine (%)	0,50	0,48
Methionine + Cysteine (%)	0,90	0,85
Calcium (%)	0,90	0,80
Available phosphorus (%)	0,45	0,42
Metabolizable energy (Kcal/Kg)	3.100	3.200

### Growth Performance

Each bird was weighed individually from day one and at the end of each week (d 1, 7, 14, 21, 28, and 35). The difference in the weight per replica (between weeks) was determined as the weekly weight gain. The feed consumption of each replica was recorded daily by subtracting the weight of the residual feed from the total quantity of feed offered. After that, cumulative feed consumption was measured on a cumulative basis for each replica. The weekly feed conversion rate was calculated on a cumulative basis for each replica. The values of each replica were calculated based on the weekly average live weights and the average weekly feed consumption. At the end of the experimental period (35 d), the European efficiency factor (**EEF**) was calculated for each replica, based on the age of broilers at sacrifice, their average live weight, viability, and the feed conversion rate. Using the following formula:

$$\text{EEF}=\frac{\text{Viablility}\;(\%)\;\times \;\text{live}\;\text{weight}\;(\text{Kg})\;\times \;100}{\text{Age}\;\text{at}\;\text{sacrifice}\;\times \;\text{feed}\;\text{conversion}\;\text{rate}}$$

### Fecal Bacterial Counts

Fresh faucal samples were collected from the bed of each replica at the end of each week (d 7, 14, 21, 28, and 35). The samples were analyzed at the Bacteriology Laboratory of the School of Veterinary Medicine of the National University of Costa Rica, Heredia, Costa Rica. Bacterial counts of all samples were determined by the plate count method. Samples were serially diluted (10^−1^ to 10^−17^) in Buffer Peptone Water and plated in specific selective culture media for *E. coli, Enterococcus* spp.*,* and *Lactobacillus* spp. following the methodology suggested by the media manufacturer. To quantify *E. coli,* samples were cultured on Petrifilm *E. coli*/Coliform cards (3M) for 48 h ± 2 h at 35°C ± 1°C. For *Enterococcus,* samples were cultured on Kanamycin Aesculin Azide Agar (Oxoid) at 42°C ± 0.3°C for 18 to 24 h. For *Lactobacillus,* samples were cultured on Rogosa Agar (Oxoid) and incubated under microaerophilic conditions at 35°C ± 1°C for 72 h. Colony-forming unit (**CFU**) counts were performed by selecting blue colonies with gas for Petrifilm *E. coli*/Coliform, small white-gray colonies for Rogosa Agar, and black colonies for Kanamycin Aesculin Azide Agar. The colonies were counted after the incubation periods, and the values were expressed as log10 CFU/gram of feces. The rate of change (**RC**) was calculated using the mean value (X̄) (CFU/gram) of the CO group (for each bacteria type) as value 1. The individual experimental values (CFU/gram) of each group (CO, AGP and PB) were divided against the mean value of the CO group using the following formula:

$$\text{RC}=\frac{\mathrm{Indiv}.\mathrm{Exp}.\mathrm{Value}(\mathrm{CFU}/\mathrm{gram})}{\bar{X}\mathrm{CO}\mathrm{group}(\mathrm{CFU}/\mathrm{gram})}$$

### Hemogram Analysis

At the end of wk 5 (35 d), 3 birds were randomly selected per replica. Blood samples were collected in heparinized tubes by puncturing the brachial vein for a complete hemogram analysis. Blood samples were analyzed at the Laboratory of Clinical Analysis of the School of Veterinary Medicine of the National University of Costa Rica, Heredia, Costa Rica, following standard protocols for avian blood samples.

### Villus Histomorphometry

At the end of wk 5 (35 d), 3 birds per replica were selected at random, sacrificed by cervical dislocation, and a necropsy was performed. Samples were analyzed at the Avian Pathology Laboratory of the School of Veterinary Medicine of the National University of Costa Rica, Heredia, Costa Rica. About 2 sections (of 2 centimeters in size) were collected per sample (per bird) for the histomorphometric study from the anterior portion of the small intestine (descending portion, 5 centimeters after the duodenal loop).

The intestinal tissues were excised, emptied of chyme, and then fixed with 4% paraformaldehyde solution. The intestinal segments were dehydrated in an ascending gradient of ethanol. These samples were then cleaned in xylene, embedded in paraffin wax, processed into slices, stained with hematoxylin and eosin (Zhang et al., 2021), and observed under a light microscope. Ten villi in each sample (2 sections) were randomly selected (30 villi per replicate; therefore 150 villi per treatment) and measured using an Olympus trinocular microscope BX53, DF73 digital camera, and CellSens Entry CS Photography Program. The mucosa length was determined from the muscular layer of the mucosa to the lumen of the organ. For villi, length was taken from the tip of the villus to the bottom. In addition, crypt depth measurements were taken from the base of the villus to the submucosa. Measurements were made using the micron scale (μm); the values were tabulated in averages (Pelicano et al., 2005; Rajput et al., 2013; Rodríguez and Moreno, 2016).

### Statistical Analysis

All data were presented as the mean with pooled SEM values. Statistical analyses were carried out with InfoStat (FCA-UNC., Cordoba, AR). One-way ANOVA followed by Tukey test was used to evaluate the differences among the treatment groups, with block and treatment as fixed effects, to establish differences between feeding treatments. Data transformation was performed for normality when variances were not homogeneous (Steel et al., 1997). The normality of the data sets was evaluated by testing residuals using the Anderson-Darling test. Statistical trends were similar for both transformed and untransformed data; therefore, the untransformed means and the SEM are shown. A *P*-value less than 0.05 was considered statistically significant. This trial was randomly divided into 3 treatments, with 5 replicates per group and ten chickens per replicate. A randomized complete blocks design with 5 blocks and 3 treatments per block was used. The statistical model for randomized design was Y_ij_= µ + T_i_ + ß_j_ + Ɛ_ij_. Y_ij_ represents the observation for the dependent variables at the j^th^ replicate in the i^th^ treatment (i = 1 to 3), µ is the overall mean, T_i_ is the treatment effect i, ß_j_ is the block effect j (j = 1 to 5) and Ɛ_ij_ is the random residual error. The mortality was estimated using the Kaplan-Meier estimation method.

### Ethics

All procedures were approved by the Bioethics and Animal Welfare Commission of the School of Veterinary Medicine of the National University of Costa Rica (UNA-EMV-CBBA-ACUE-005-2019).

## RESULTS

### Production Performance

The results of the productive performance are shown in Tables 2–4. The body weight did not present significant differences (*P* > 0.05) during the first 2 wk of the study. However, at the end of the third week (d 21), a significant difference (*P* ˂ 0.05) was observed between the experimental groups (AGP and PB) and the control group. No difference (*P* > 0.05) was observed between the AGP and PB group. This difference was maintained until the end of the study (d 35). Feed consumption did not show differences (*P* > 0.05) during the first 3 wk. However, at the end of wk 4 (d 28) and 5 (d 35), significant statistical differences (*P* ˂ 0.05) were observed between the 2 experimental treatments (PB and AGP) and CO. There was no difference (*P* > 0.05) between AGP and PB groups.

**Table 2:** Effects of dietary treatment on body weight of broilers.

Treatment^1^	D 1	D 7	D 14	D 21	D 28	D 35
CO	46.76	133.02	339.02	791.48^b^	1403.90^b^	2132.92^b^
AGP	46.36	134.48	357.49	828.22^a^	1470.80^a^	2205.00^a^
PB	45.88	139.76	374.38	831.02^a^	1456.30^a^	2215.92^a^
SEM	3.72	16.57	59.11	60.18	69.86	72.05
*P*-value	0.499	0.113	0.111	0.031	0.044	0.039

Values are expressed as means with pooled SEM values, n = 150.

a,b Means with different superscripts in the same column differ (*P* ˂ 0.05).

1 Abbreviations: AGP, antibiotic growth promoter; CO, control; PB, probiotic.

The feed conversion calculated at the end of each week did not show differences (*P* > 0.05) during the first weeks of the study. However, at the end of wk 4 (d 28) and 5 (d 35), values with significant differences (*P* ˂ 0.05) were obtained, was significantly reduced in both AGP and PB groups compared CO group. The European efficiency factor obtained at the end of the trial (d 35) were 404.29, 423.84, and 425.08 (CO, AGP, and PB, respectively). These results showed significant statistical differences between the 2 experimental treatments and the control (SEM 20.07, *P*-value 0.036). No significant differences were observed between the experimental treatments (AGP vs. PB).

### Hemogram Analysis

The hemogram analysis results are shown in Table 5. No statistically significant differences were observed for any parameter in the red or white cell counts.

### Fecal Bacterial Counts Over Time

**Figure 1:**
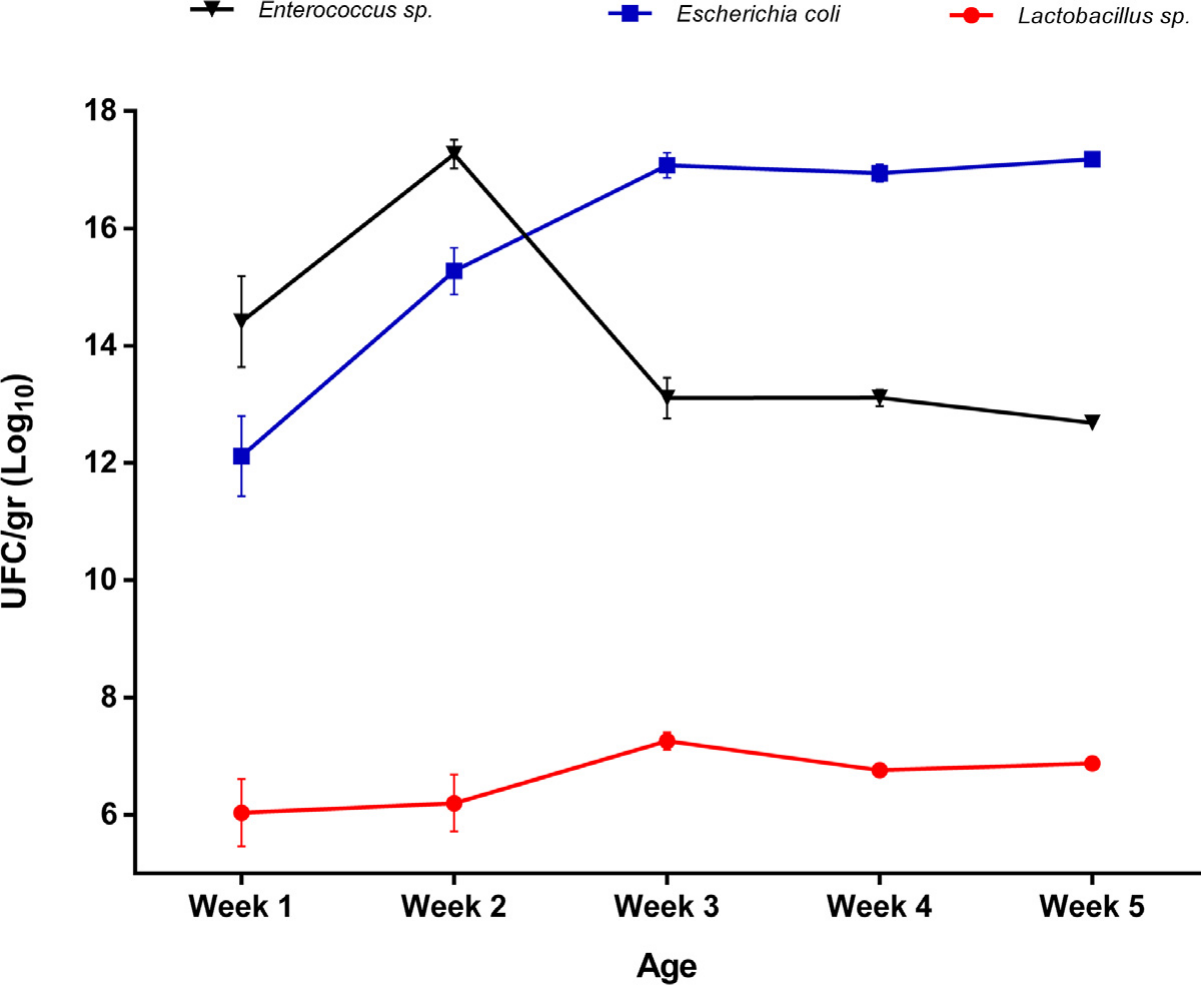
Fecal counts of *Enterococcus* spp., *Escherichia coli* y *Lactobacillus* spp. over time (UFC/g Log_10_) in broiler chickens fed control diet for five weeks.

**Figure 2:**
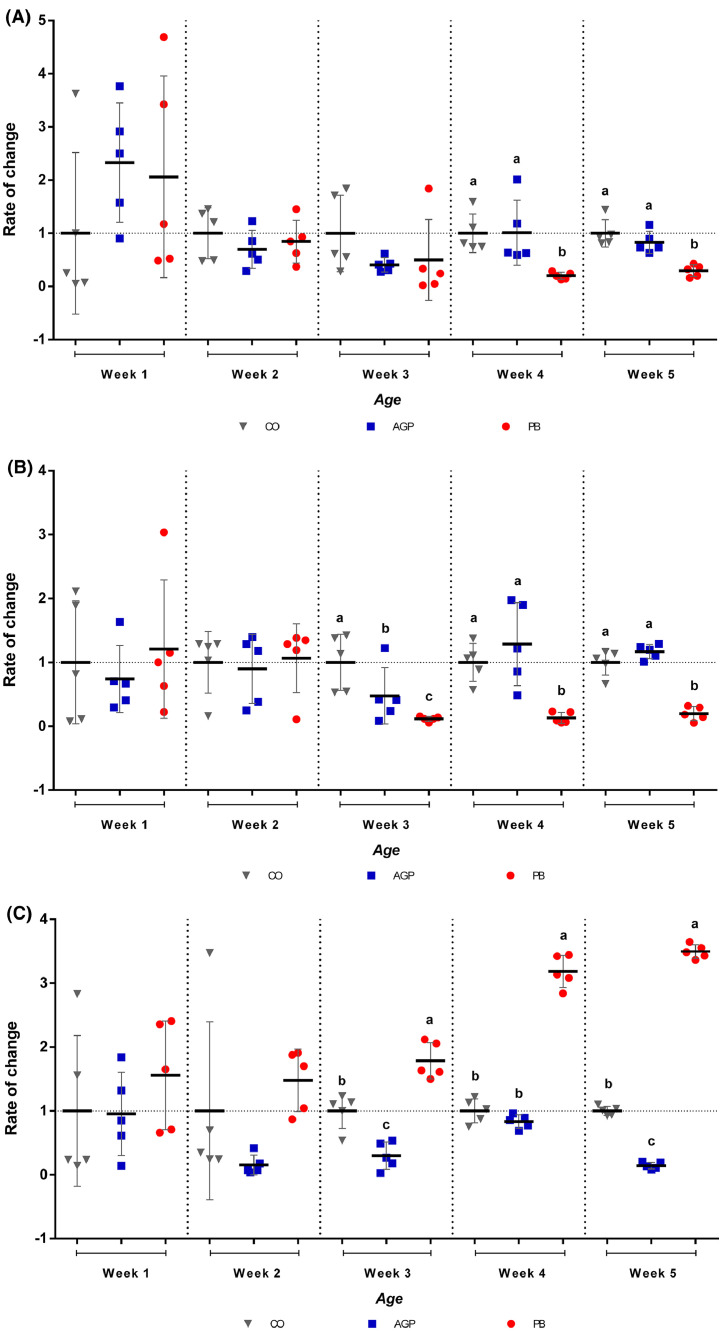
Effect of dietary treatment diets on fecal (A) *Enterococcus* spp., (B) *Escherichia coli,* and (C) *Lactobacillus* spp. population (rate of change). ^a,b,c^ Different letters mean significant differences between the groups (*P* ˂ 0.05), n = 15. Abbreviations: AGP, antibiotic growth promoter; CO: control; PB, probiotic.

Fecal bacterial counts of the control group are shown in Figure 1. *Enterococcus* spp. counts decreased from the second week onwards. Contrarily, *E. coli* counts increased at the beginning of the trial, reaching a stationary phase. *Lactobacillus* spp. counts were lower when compared to the other bacteria and remained stable over time. The rates of change of PB and AGP counts against the control group (CO) are shown in Figure 2. It was observed that from the end of wk 4 and until the end of the trial, *Enterococcus* spp. counts were significantly lower (*P* ˂ 0.05) in the PB group. No difference was observed between CO and AGP groups at this time point (Figure 2A). Besides, *Escherichia coli* counts were also significantly lower (*P* ˂ 0.05) in the PB group from the third week of the trial until the end (Figure 2B). *Lactobacillus* spp. counts were significantly higher (*P* ˂ 0.05) in the PB group from the third week until the end of the study. Two statistical differences were also found between CO and AGP groups. First, it was observed that *E. coli* counts were significantly lower (*P* ˂ 0.05) in the AGP group during the third week, and second, *Lactobacillus* spp. count was significantly higher (*P* ˂ 0.05) counts during the third and fifth weeks in the CO group.

### Villus Histomorphometry

The histomorphometry measurement of the anterior portion of the broiler's intestine is shown in Table 6. The height of the mucosa and the height of the villi were significantly higher (*P* ˂ 0.05) in the PB group, contrary to the thinnest mucosa observed in the AGP group, and intermediate values were obtained in CO. No significant differences (*P* > 0.05) were observed in measuring the thickness of the villi. The depth of the crypts was significantly lower (*P* ˂ 0.05) in the PB group, compared to the other 2 groups (CO and AGP). No significant difference (*P* > 0.05) was observed between CO and AGP groups concerning the depth of the crypts.

## DISCUSSION

The current results indicate that *B. subtilis* QST713 (used as a probiotic) positively affected productive performance, animal health, and intestinal integrity. Thus, our results show that probiotics such as *B. subtilis* are a viable and natural alternative for replacing growth-promoting antibiotics such as BMD in Cobb broilers.

When analyzing zootechnical parameters to determine the effects of the probiotic *B. subtilis* (QST713) as an alternative to BMD, there were no treatment effects on body weight, consumption, feed conversion, and EEF between groups from wk 1 to wk 3. Likewise, a similar production performance was observed between PB and AGP groups at both 28 and 35 d of age (Table 2, Table 3, Table 4), significantly higher (*P* ˂ 0.05) than the CO group. Our results resemble those reported by other researchers using other *B. subtilis* strains (Sikandar et al., 2017; Teo and Tan, 2007; Park et al., 2020; Zhang et al., 2021). Moreover, in our trial, EEF results were similar between AGP and PB groups, which is highly relevant because it includes all indicators of zootechnical importance and is in agreement with previous results (Fritts et al., 2000; Opalinski et al., 2007; Bitterncout et al., 2011; Knap et al., 2011; Jayaraman et al., 2017; Manafi et al., 2017; Krueger et al., 2020). While other reported a significant increase in the productive performance using *B. subtilis* as a probiotic (Aliakbarpour et al., 2012; Barrera et al., 2014; Nguyen et al., 2015; Harrington et al., 2016; Abudabos et al., 2017; Ciurescu et al., 2020; Sandvang et al., 2021), while others, reported null or insignificant productive effects (Pelicano et al., 2004; Lee et al., 2010; Dersjant et al., 2014; Waititu et al., 2014). These differences can be due to factors inherent in each experimental design, such as the combination of probiotic strains, administration dose, age of the birds, farm hygiene, environmental stress factors, and diet composition (Aliakbarpour et al., 2012; Bai et al., 2017).

**Table 3:** Effects of dietary treatment on cumulative feed consumption of broilers.

Treatment^1^	D 7	D 14	D 21	D 28	D 35
CO	114.70	445.96	1081.48	2135.64^b^	3204.92^b^
AGP	115.14	453.80	1112.64	2170.96^a^	3274.54^a^
PB	119.00	475.84	1123.96	2180.56^a^	3291.68^a^
SEM	7.76	44.68	71.22	90.46	100.12
*P*-value	0.661	0.583	0.654	0.0424	0.0432

Values are expressed as means with pooled SEM values, n = 15.

a,b Means with different superscripts in the same column differ (*P* ˂ 0.05).

1 Abbreviations: AGP, antibiotic growth promoter; CO, control; PB, probiotic.

**Table 4:** Effects of dietary treatment on cumulative feed conversion of broilers.

Treatment^1^	D 7	D 14	D 21	D 28	D 35
CO	0.86	1.31	1.37	1.52^a^	1.51^a^
AGP	0.86	1.27	1.35	1.48^b^	1.49^b^
PB	0.85	1.27	1.35	1.49^b^	1.49^b^
SEM	0.03	0.05	0.06	0.04	0.04
*P*-value	0.923	0.353	0.724	0.047	0.048

Values are expressed as means with pooled SEM values, n = 15.

a,b Means with different superscripts in the same column differ (*P* ˂ 0.05).

1 Abbreviations: AGP, antibiotic growth promoter; CO, control; PB, probiotic.

Our results reached statistical significance in the third and fourth weeks, which agrees with previous reports (Fritts et al., 2000; Jacquier et al., 2019; Bilal et al., 2021). However, other reports (Molnar et al., 2011; Gadde et al., 2017) found significant differences from the first and second weeks. These differences should be further investigated since many different mechanisms have been reported to affect bird development. For instance: decrease in intestinal pH (Reis et al., 2017; Ciurescu et al., 2020), reduction of toxic compounds (Aliakbarpour et al., 2012), competitive exclusion (Abudabos et al., 2017; Bai et al., 2017), mucin production (Bilal et al., 2021), modulation of the immune system (Camargo et al., 2012), production of antibacterial substances (Darabi et al., 2014, Batkowska et al., 2015, Florido et al., 2017), fermentation of simple sugars (Barrera et al., 2014, Sandvang et al., 2021), production of lactic acid (Barrera et al., 2014) and production of extracellular enzymes such as amylase, protease, and lipase (Ciurescu et al., 2020).

PB showed no significant impact on the hematological profile of broilers when compared with the other experimental groups (Table 5). All the results were in ranges expected for birds in good health status (Avilez et al., 2014; Diaz et al., 2016). Our results agree with previous reports showing that probiotics do not induce significant changes in the hematological values of the broilers. (Park and Kim 2014; Gutierrez and Corredor 2017; Park et al., 2018). Nevertheless, normal blood counts do not exclude the possibility of altered immune status or stress induced by environmental factors (Avilez et al., 2014; Diaz et al., 2016).

**Table 5:** Effect of dietary treatment on blood count of broiler chickens at the end of experimental diets on broilers at 5 wk of age.

	Treatment^1^				
Parameter	CO	AGP	PB	SEM	*P*-value
Hematocit (%)	27.07	28.33	28.27	2.90	0.413
Hemoglobin (g/dL)	8.98	9.15	9.45	0.83	0.290
MCHC (g/dL)^2^	33.33	32.27	33.20	2.41	0.426
Leukocytes (uL)	4814.93	5448.67	5292.67	1165.89	0.306
Neutrophils (%)^3^	60.67	64.93	65.13	9.73	0.373
Eosinophils (%)	3.46	3.23	5.00	2.85	0.231
Basophils (%)	3.00	3.10	3.31	2.44	0.957
Lymphocytes (%)	34.27	30.27	27.40	8.93	0.105

Values are expressed as means with pooled SEM values, n = 45.

^a,b^Means with different superscripts in the same column differ (*P* ˂ 0.05).

1 Abbreviations: AGP, antibiotic growth promoter; CO, control; PB, probiotic.

2 Mean corpuscular hemoglobin concentration.

3 Segmented neutrophils.

**Table 6:** Effect of dietary treatment on intestinal morphology (anterior portion) of broilers at 35 days of age.

Treatment^1^	Mucosal height (µm)	Villus height(µm)	Villus width (µm)	Crypt Depth (µm)
CO	868.06^b^	740.46^b^	130.80	93.20^a^
AGP	760.29^c^	649.06^c^	131.55	93.94^a^
PB	1018.43^a^	878.46^a^	132.79	86.84^b^
SEM	10.888	9.958	1.854	1.098
*P*-value	˂0.0001	˂0.0001	0.906	0.014

Values are expressed as means with pooled SEM values, n = 450.

a,b Means with different superscripts in the same column differ (*P* ˂ 0.05).

1 Abbreviations: AGP, antibiotic growth promoter; CO, control; PB, probiotic.

The dynamics of the bacterial species monitored showed that the populations stabilized towards the end of the trial. PB group showed a significant decrease of *Enterococcus* spp. and *E. coli* and increased *Lactobacillus* spp. compared with the other 2 groups. In contrast, the AGP group did not show a comparative difference with the CO group in the counts of *Enterococcus* spp. and *E. coli* but showed decreased *Lactobacillus* spp*.* (Figure 2), suggesting a minor effect of AGP on potential pathogenic bacteria and a negative effect on beneficial flora.

The microbial modulation found in our trial (Figures 1 and 2) coincided with some reports (Cao et al., 2013; Jeong and Kim 2014; Forte et al., 2016; Guo et al., 2017; Ciurescu et al., 2020; Bilal et al., 2021) in that this late modulation of the bacterial populations takes approximately 2 wk to reach microbial stability in the intestine (Diaz et al., 2017). This process's relevance includes the modulation of the biosynthesis and degradation of substances and activating different signaling cascades and secretory chemical agents (Dharmani et al., 2009; Aliakbarpour et al., 2012). Some of these mechanisms reported with the use of *B. subtilis* are: 1) sustained increase in mucin secretion (Jacquier et al., 2019) (which plays a vital role in maintaining the architecture of the mucus layer on the intestinal surface); 2) significant increase in goblet cells (Camargo et al., 2012) (which directly affects the innate immune response and regulates the response to inflammation/infection); and 3) increase in the reactions of the mucous membranes to pathogens and putrefaction agents (Barrera et al., 2014).

Our results suggest that PB inhibited Gram-negative (*E. coli*) and Gram-positive bacteria (*Enterococcus* spp.) growth and stimulated *Lactobacillus* spp increase, which is similar to others trials (Forte et al., 2016; Guo et al., 2017; Park et al. 2018). Furthermore, these results are considered a highly efficient mechanism (Aziz et al., 2015; Florido et al., 2017), since the increase in *Lactobacillus* spp. is associated with an immediate consumption of oxygen by *B. subtilis* and subsequent creation of an anaerobic environment, which reduced harmful bacteria (Hoa et al., 2000: Jeong and Kim 2014; Latorre et al., 2014). Other studies did not show an increase in *Lactobacillus* spp. but did demonstrate a decreased *E. coli* counts (Molnar et al., 2011).

The increase in *Lactobacillus* spp*.* is very positive, considering its capability to bind to a specific receptor in the enterocyte and stimulate the positive regulation of mucin (Mattar et al., 2002), ferment glucose, and produce lactic acid. This activity decreases intestinal pH, hindering the reproduction and colonization of potentially pathogenic bacteria (Cao et al., 2013; Diaz et al., 2017), such as *Salmonella* spp. (Knap et al., 2011; Park and Kim 2014; Park et al., 2018) and *Clostridium perfringens* (Melegy et al., 2011; Tactacan et al., 2013).

Our results show that PB addition increased the intestinal mucosa and villi height and decreased the depth of the crypts compared to the other groups. Other authors reported similar results (Pelicano et al., 2005; Pelicano et al., 2007; Aliakbarpour et al., 2012; Chávez et al., 2016; Jayaraman et al., 2017; Jacquier et al., 2019). Furthermore, longer villi with shallow crypts increase the nutrient absorption surface and indicate a sufficiently mature and functionally active epithelium (Chavez et al., 2016). Furthermore, an increase in the villi's length is associated with greater production of enzymes, improved nutrient transport, and more quantity and size of goblet cells (Rahimi et al., 2009; Aliakbarpour et al., 2012). On the other hand, the AGP group presented the shortest mucous membranes and villi and the deepest crypts. These results are expected since bacitracin has shown to reduce the thickness of the walls, thinning the intestinal villi, and reducing mucosal cells' proliferation (Engberg et al., 2000; Sims et al., 2004; Fasina and Thanissery 2011). Contrarily, the deep and wide crypts imply a higher nutrient requirement due to increased cellular turnover for the maintenance of this tissue and, with it, a lower productive yield (Chavez et al., 2016; Jayaraman et al., 2017).

Putting together the results in bacterial dynamics and intestinal histomorphometry, we propose that bacterial changes mediated the changes in intestinal morphology. The small intestine's digestive function is closely related to the architecture of the mucosa and the structure of the villi (Aliakbarpour et al., 2012). The trophic action of *B. subtillis* can explain the changes in the villi's length because they stimulate the mitotic process in the crypt-villus region through competitive exclusion, allowing proliferation mechanisms in the intestinal mucosa (Barrera et al., 2014). All of the above ultimately translates into a healthy intestine that maximizes nutrient uptake and, consequently, a better zootechnical performance. We conclude that *B. subtillis* QST713, used at the suggested commercial dose (100 g/ton), is an effective growth-promoting alternative to BMD that modulates the microbiota and intestinal architecture, thus producing zootechnical yields consistent with BMD.
